# Evaluation of the Reliability, Utility, and Quality of Information Used in Total Extraperitoneal Procedure for Inguinal Hernia Repair Videos Shared on WebSurg

**DOI:** 10.7759/cureus.5566

**Published:** 2019-09-04

**Authors:** Abdulcabbar Kartal, Abut Kebudi

**Affiliations:** 1 General Surgery, Okan University Medical Faculty, Istanbul, TUR

**Keywords:** websurg, tep, surgical video, inguinal hernia, internet, technical quality

## Abstract

Purpose

The Internet is widely used by patients and physicians for obtaining medical information. WebSurg is a valuable information resource that can improve the learning experience of medical professionals if used appropriately. This study aimed to evaluate the quality and accuracy of videos on the total extraperitoneal procedure (TEP) for inguinal hernia repair.

Methods

We included 32 videos returned by the WebSurg search engine in response to the keyword “TEP.” Video popularity was evaluated using the video power index (VPI). The videos’ educational quality was measured using the DISCERN score, Journal of American Medical Association (JAMA) benchmark criteria, and Global Quality Score (GQS). Technical quality was measured using the TEP Scoring System (TepSS) by three surgeons who performed TEP routinely.

Results

All videos were obtained from medical doctors; 12.5% of the videos were uploaded from Belgium; 3.1%, China; 6.3%, Colombia; 6.3%, England; 59.4%, France; 9.4%, Germany; and 3.1%, Korea. No significant differences were noted in terms of the VPI, DISCERN scores, JAMA benchmark criteria, GQSs, and TepSS scores (p > 0.05). The mean VPI, DISCERN score, JAMA benchmark criteria, GQS, and TepSS score were 9454.53 ± 15085.57, 32.75 ± 6.99, 2.31 ± 0.47, 1±0, and 9.25 ± 2.36, respectively. No significant associations were noted between the VPI and DISCERN score, JAMA benchmark criteria, and GQS (p > 0.05). Similarly, there was no significant association between the VPI and TepSS scores (r = 0.100; p = 0.587).

Conclusions

The online information on TEP is of suboptimal quality. Although limited information is available on preoperative and postoperative processes, the educational potential of WebSurg cannot be ignored.

## Introduction

Toward the end of the 19th century, McVay and Bassini described the detailed pathological anatomy of the inguinal canal and developed surgical techniques for appropriate inguinal hernia repair. Although several repair methods have been applied to treat inguinal hernia, concerns regarding the complications associated with inguinal hernia recurrence cannot be ignored, thereby requiring leading surgeons to explore different methods. The most interesting of these methods, which have recently become popular, are minimally invasive ones [1-2]. Minimally invasive surgical methods are becoming increasingly popular owing to the associated low postoperative pain and the possibility for rapid return to daily activities. Laparoscopic inguinal and femoral hernia repair can be safely performed. Laparoscopic approaches were first used in 1992 to treat inguinal hernias [3]. Transabdominal preperitoneal and total extraperitoneal methods have been established as the current laparoscopic approaches for hernia repair.

With the rapid spread of the Internet worldwide, websites offering different content and information have also become a part of our lives. Written and visual data available on these websites are extremely important, as these websites provide information to both general surgeons and patients. With advancements in surgical technologies, accessing such concerns has become particularly important for surgeons. YouTube®, TVASURG, and WebSurg are the most well-known websites, and some of these are open-access, video-sharing websites allowing content sharing and viewing at the academic level [4]. Due to their widespread use and the ease of Internet access, these websites have become a reference source for health-related information. However, these sites may also provide insufficient, biased, or incorrect information owing to the abundance and diversity of authors providing content to the websites and the difficulty or impossibility of conducting a healthy peer-review process. WebSurg, IRCAD’s online university and a representative of the Web 2.0 phenomenon, was launched by Professor Jacques Marescaux and his team at the European Institute of TeleSurgery in 2000 in Strasbourg. WebSurg provides information on minimally invasive surgical procedures within the scope of continuous medical education.

Here, we aimed to evaluate the popularity of the videos in terms of their quality and accuracy using the recognized quality scoring systems: DISCERN score, Global Quality Score (GQS), Journal of American Medical Association (JAMA) benchmark criteria, and video power index (VPI). These systems have been created to assess both the videos’ views and like ratios. The TEP Scoring System (TepSS) has been designed for a more detailed assessment of WebSurg videos in terms of the total TEP-specific diagnoses, classification, treatment alternatives, and complications.

## Materials and methods

Inclusion and exclusion criteria

The videos returned by WebSurg in response to the keyword “TEP” between January 1, 2019, and February 15, 2019, were included. A total of 32 videos titled “surgical intervention” were included in the study, whereas those titled “lecture (n = 7),” “expert opinion (n = 8),” and “operative technique (n = 3)” were excluded. Lecture, expert opinion, and operative technique videos are similar and might contain content different from that of surgical intervention videos; further, these types of videos are of high educational value to surgical trainees. To avoid confusion and make a full assessment, these types of videos were excluded.

All videos were evaluated in terms of popularity, quality of training, and surgical technique. The educational quality and accuracy of the video content were evaluated using the DISCERN score, JAMA benchmark criteria, and GQS. TepSS was also used to evaluate the medical and technical quality of the information.

TepSS

**Figure 1:**
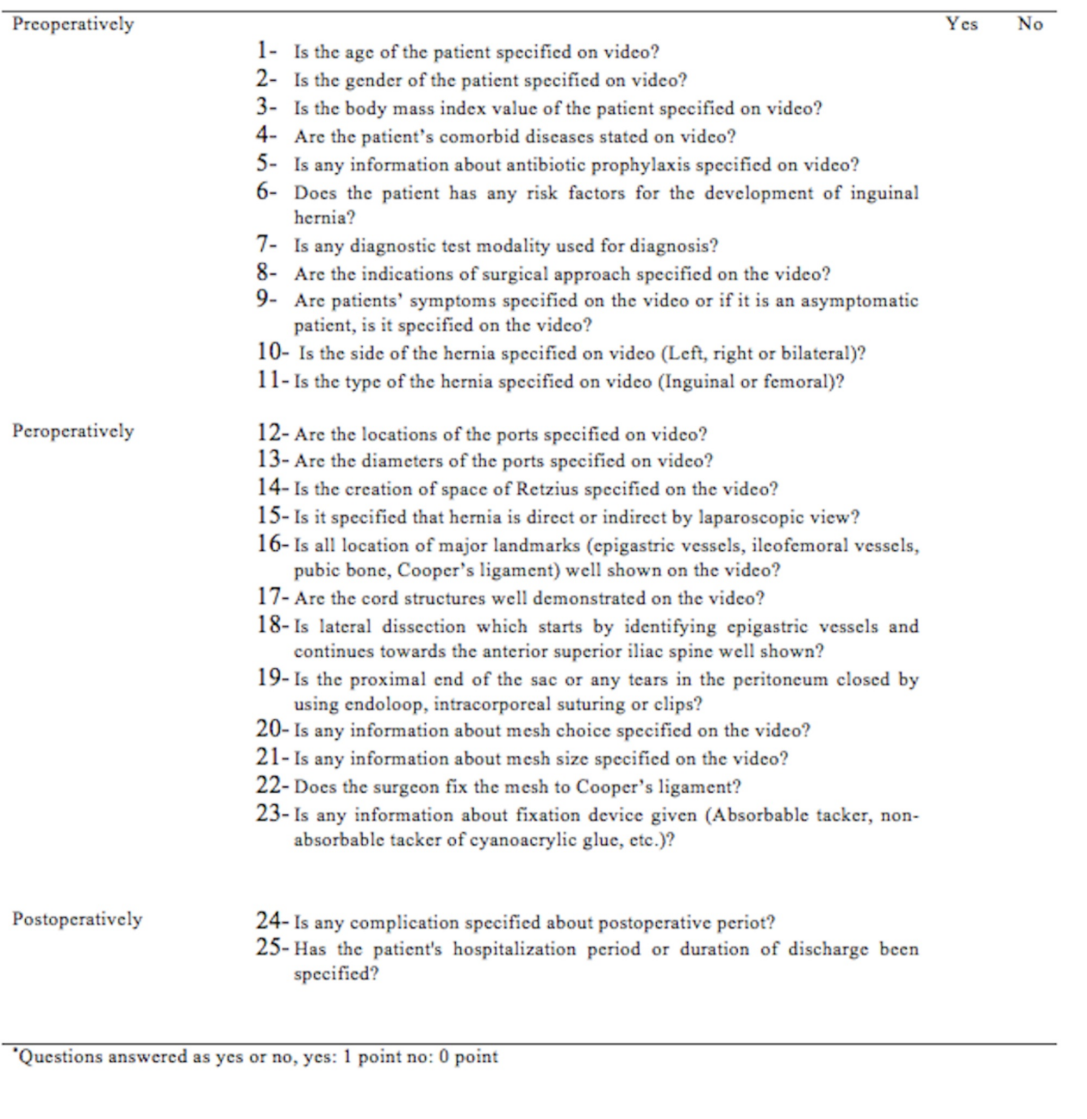
TEP Scoring System

This is a specific scoring system for assessing the preoperative, perioperative, and postoperative technical quality of TEP videos. It was used by three expert surgeons with education on laparoscopic inguinal hernia surgery from different training and research hospitals and who performed a high number of TEPs in daily practice (Figure 1).

The scores were evaluated by the authors on the basis of the European Hernia Society’s International Guidelines for groin hernia management [5]. This scoring system comprised 25 questions and three sections. One point was given for each question whose reply was available in the audio or text of the video. Using K-means clustering, video quality was classified according to the TepSS scores as follows: poor (TepSS score < 6.64), suboptimal (TepSS score between 6.64 and 10.62), and good (TepSS score > 10.62) quality.

Video power index

To assess both the videos’ views and like ratios (popularity), the VPI, which was first described by Erdem MN et al., was calculated using the following formula: VPI = like ratio × view ratio / 100 [6].

DISCERN questionnaire

To determine the quality of the information and the offered treatment choices, the DISCERN questionnaire, which was developed by professionals at Oxford University in the United Kingdom, was used [7]. This questionnaire has a scoring range of 0-80 points and three sections comprising 16 questions. Each question is rated on a five-point scale ranging from “no” to “yes” (80: the quality criterion has been completely fulfilled; 32-64: the quality criterion has been partially fulfilled; 16: the quality criterion has not at all been fulfilled).

GQS

This five-point scale described by Bernard et al. was used to assess the educational value of each video (1: poor quality, very unlikely to be of any use to patients; 2: poor quality but some information of very limited use to patients present; 3: suboptimal flow, some information covered but important topics missing and somewhat useful to patients; 4: good quality and flow, most important topics covered and useful to patients; and 5: excellent quality and flow and highly useful to patients) [8].

JAMA benchmark criteria

The transparency and publication information of each video were evaluated according to the JAMA benchmark criteria, with scores ranging from 0 to 4, as suggested by Silberg et al. [9].

Statistical analysis

The Number Cruncher Statistical System (NCSS) 2007 (Kaysville, Utah, USA) software was used for statistical analysis. Descriptive statistical methods (mean, standard deviation, median, first quarter, third quarter, frequency, percentage, minimum values, and maximum values) were used to evaluate the study data. Normal distribution of quantitative data was tested using the Shapiro-Wilk test and graphical investigations. The independent groups' t-test was used to compare quantitative variables showing normal distribution, whereas the Mann-Whitney U-test was used to compare quantitative variables not showing normal distribution. The Kruskal-Wallis and Dunn-Bonferroni tests were used to compare multiple groups of quantitative variables not showing normal distribution. Spearman’s correlation analysis was used to evaluate the relationships among the quantitative variables. To classify the video quality according to the TepSS scores, K-means clustering was used. p < 0.05 was considered statistically significant.

## Results

Examination of the educational quality of the videos revealed that most videos were uploaded by medical doctors (MDs) and that France uploaded the greatest number of videos (Table 1).

**Table 1:** Information on descriptive properties FACS: Fellow, American College of Surgeons; MD: Doctor of Medicine; MRCS: Membership of the Royal Colleges of Surgeons of Great Britain and Ireland

		n	%
Academic Degree	FACS	3	9.4
	MD	28	87.5
	MRCS	1	3.1
Country	Belgium	4	12.5
	China	1	3.1
	Colombia	2	6.3
	Britain	2	6.3
	France	19	59.4
	Germany	3	9.4
	Korea	1	3.1
		Min-Max	Mean±sd (Median)
Publication duration (days)		257-6039	3520.59±1566.98 (3624)
Duration of the videos (seconds)		242-1646	851.31±343.51 (841)
Number of views (n)		843-24288	6660.66±5094.33 (5151.5)
Number of likes (n)		24-583	114.44±129.99 (66)

Other descriptive characteristics related to the videos are summarized in Table 2. 

**Table 2:** Information on scores VPI: video power index; JAMA: Journal of American Medical Association; TEP: total extraperitoneal procedure

	Min-Max	Mean±sd (Median)
VPI	295-59182 (3266)	9454.53±15085.57
DISCERN Score	20-46 (32.5)	32.75±6.99
JAMA Benchmark	2-3 (2)	2.31±0.47
Global Quality Score	1	1
TEP Score	3-14 (9.5)	9.25±2.36

When the scores of the videos were examined, the following mean values were noted: VPI, 9454.53 ± 15085.57; DISCERN score, 32.75 ± 6.99; JAMA benchmark criteria, 2.31 ± 0.47, GQS, 1; and TepSS score, 9.25 ± 2.36 (Table 3).

**Table 3:** Comparison of scores according to descriptive characteristics FACS: Fellow, American College of Surgeons; MD: Doctor of Medicine; MRCS: Membership of the Royal Colleges of Surgeons of Great Britain and Ireland; VPI: video power index; JAMA: Journal of American Medical Association; GQS: Global Quality Score; TEP: total extraperitoneal procedure

	VPI	Discern Score	JAMA Benchmark	GQS	TEP Score
Academic Degree					
FACS	4358 (843, -)	34 (26, -)	2 (2, -)	1 (1, 1)	10 (9, )
MD	3266 (1264.5, 10789.5)	32 (28, 37)	2 (2, 3)	1 (1, 1)	9,5 (7, 11)
MRCS	2258 (2258, 2258)	44 (44, 44)	3 (3, 3)	1 (1, 1)	7 (7, 7)
p	0.860	0.276	0.328	1.000	0.327
Country					
Belgium	3222.5 (3041.75, 43452.5)	29 (23.5, 39.75)	2.5 (2, 3)	1 (1, 1)	9 (4, 11.75)
China	59182 (59182, 89182)	28 (28, 28)	3 (3, 3)	1 (1, 1)	11 (11, 11)
Colombia	3954 (843, -)	30 (26, -)	2 (2, 2)	1 (1, 1)	12 (10, -)
Britain	1276.5 (295, -)	38 (32, -)	2.5 (2, -)	1 (1, 1)	8 (7, -)
Franca	3221 (1147, 7011)	35 (28, 40)	2 (2, 3)	1 (1, 1)	10 (8, 11)
Germany	5392 (1888, -)	31 (25, -)	2 (2, 2)	1 (1, 1)	7 (7, -)
Korea	20047 (20047, 20047)	37 (37, 37)	2 (2, 2)	1 (1, 1)	9 (9, 9)
p	0.312	0.683	0.455	1.000	0.402
Total	3266 (1264.5, 7051.5)	32.5 (28, 38.5)	2 (2, 3)	1 (1, 1)	9.4 (7, 11)

No significant difference was noted among the VPI, DISCERN score, JAMA benchmark criteria, GQS, and TepSS score according to the academic degree and the country of video origin (p > 0.05) (Table 4).

**Table 4:** Determination of the relationship between quantitative variables and scores VPI: video power index; JAMA: Journal of American Medical Association; TEP: total extraperitoneal procedure

		VPI	Discern Score	JAMA Benchmark	TEP Score
Publication duration	r	-0.469	-0.227	-0.230	-0.169
	p	0.007**	0.211	0.205	0.354
Duration of the videos	r	0.351	0.419	0.263	0.095
	p	0.049*	0.017*	0.146	0.604
Views	r	0.780	0,393	0,453	-0,013
	p	<0.001***	0.026*	0.009**	0.945
Like	r	0.808	0.035	0.241	0.088
	p	<0.001***	0.850	0.184	0.632

There was a negative association between the video durations and VPI (r = −0.469; p = 0.007), whereas no significant association was noted between the DISCERN score, JAMA benchmark criteria, and TepSS score (p > 0.05). A positive correlation was observed between the video durations and the VPI (r = 0.351; p = 0.049) and DISCERN scores (r = 0.419, p = 0.017), whereas no significant correlation was noted between the JAMA benchmark criteria and TepSS score (p > 0.05). There was a positive correlation between the number of video views and the VPI (p < 0.001), DISCERN scores (p = 0.026), and JAMA benchmark criteria scores (p = 0.009), whereas there was no significant association between the number of video views and the TepSS scores (p > 0.05). There was a positive correlation between the number of likes given to the videos and the VPI (p < 0.001), whereas there was no significant relationship between the number of likes given to the videos and the DISCERN score, JAMA benchmark criteria, and TepSS score (p > 0.05; Table 5). Similarly, no significant correlation was noted among the TepSS score, VPI, DISCERN score, and JAMA benchmark criteria score of the videos (p > 0.05).

**Table 5:** Determination of the relationship between scores VPI: video power index; JAMA: Journal of American Medical Association; TEP: total extraperitoneal procedure

		VPI	Discern Score	JAMA Benchmark	TEP Score
VPI	r	1.000			
	p	-			
Discern Score	r	0.271	1.000		
	p	0.133	-		
JAMA Benchmark	r	0.372	0.095	1.000	
	p	0.036*	0.605	-	
TEP Score	r	0.100	0.163	-0.063	1.000
	p	0.587	0.371	0.732	-

## Discussion

Obvious difficulties associated with learning and practicing the theoretical and practical aspects of newly developing, minimally invasive methods as compared with traditional surgical techniques, including hernia surgery (the core procedure), are encountered during surgical education. Thus, the learning and teaching of innovative surgical techniques tend to continue even after assistantship training. General surgeons interested in this field are keen to learn these surgical techniques online and from various training boxes, courses, and conferences. As per a review of various procedures, including laparoscopic hernia repair, laparoscopic box training reportedly improves patient outcomes (e.g., length of stay), operative time, and overall performance [10]. WebSurg offers laparoscopic and endoscopic surgical videos that contribute to surgical training and a large number of educational surgical videos from different countries, making it the first website in the scope of continuous surgical training. When site statistics were examined, it was observed that the number of members increased by 1980%, that of visitors increased by 740%, and video views increased by 3300% between 2004 and 2010, indicating that this virtual university attracts surgeons’ attention [11].

Inguinal hernia repair is currently one of the most frequently performed surgeries by general surgeons [12]. Every year, approximately 20 million hernias are repaired and many surgical techniques are being attempted to overcome the complications of inguinal hernia. The main reason underlying the development of new techniques is to reduce hernia recurrence [5]. Laparoscopic hernia repair has recently started gaining importance in the surgical literature and is, therefore, beginning to influence our traditional views on inguinal hernia. Since the last two decades, laparoscopic inguinal hernia repair is being increasingly performed by senior general surgery residents in the US [13]. The use of laparoendoscopic surgery for groin hernia varies from 0% to 55% among countries. The average use of this surgery in high-resource countries is as follows: Australia, 55%; Switzerland, 40%; the Netherlands, 45%; and Sweden, 28% [14-15]. Laparoscopic hernia repair, which is performed either via the transabdominal preperitoneal (TAPP) or total extraperitoneal (TEP) approach, is gaining popularity because of its short recovery period, the possibility for rapid return to daily activities and work, and low postoperative complications. High recurrence (25%) and complication rates, which were reported when the technique was newly established, have begun to decrease owing to increasing experience and tension-free mesh hernia repair becoming prevalent [16-17]. At the beginning of their careers, general surgeons are more willing to perform laparoscopic inguinal hernia repair because they are more familiar with laparoscopic procedures during surgical training at earlier ages. Ferhatoğlu et al. reported that the willingness to perform laparoscopic inguinal hernia surgery was lower among general surgeons with ≥10 years of experience than among those at the beginning of their careers [18].

In our study, most videos were uploaded by MDs, and the country that uploaded the greatest number of videos was France. The fact that the videos are uploaded by academic professionals and published after academic review by WebSurg indicates that the quality and reliability of education may be high. Moreover, published studies have stated that videos uploaded by health professionals are of high quality and are reliable [19-20]. However, in our study, no significant associations were observed between the VPI, DISCERN score, JAMA benchmark criteria, GQS, and TepSS score according to the academic degree and country of video origin. Although this result leads to questions on whether WebSurg is adequate for laparoscopic inguinal hernia education, the fact that all videos were uploaded by academic professionals might have contributed to the lack of a significant difference.

No significant association was noted between video popularity and the educational and surgical technical video quality in our study. Similarly, considering the quality of the preoperative, perioperative, and postoperative surgical techniques in the videos, the TepSS score was low and independent of the VPI, DISCERN score, JAMA benchmark criteria, and GQS, suggesting that the videos are not sufficient for training new surgical specialists, particularly if they have not received TEP education during their residency program. This is because laparoscopic hernia repair requires special skills and a certain learning curve and because the operation time is relatively longer. The reasons for this are anatomical difficulties, the inadequacy of the perception of depth and sense of touch, and the limited range of movement.

Although it may vary according to the training center experience, no consensus has been reached with respect to learning curves in laparoscopic hernia surgery [12]. In their study involving 90 cases, Lim et al. reported that at least 30 cases are necessary to learn TEP [21]. Choi et al. evaluated 700 cases and reported 60 cases as the minimum number required for a learning curve to be 60 [22]. In the European Hernia Society’s guide, the interval of the number of cases necessary for the learning curve of a surgeon was defined as 50-100 [23].

There was a positive association among the video publication duration, video duration, and video popularity; in contrast, there was no significant association between the educational and surgical technical quality scores. This suggests that from the time TEP was first applied to this day, there has been no revision or improvement in the video contents, and this should be considered a negative point.

There was a positive correlation among the number of views, number of likes, and popularity of the videos; in contrast, no significant association was observed among the TepSS scores. The TepSS scores were not significantly higher among the videos with a higher number of likes, views, and VPIs. This indicates that even popular videos are not sufficient in terms of TEP education.

Because TEP is a very specific inguinal hernia surgery type, there are several key points that should be highlighted in the videos to make them useful for teaching the technique. In the preoperative period, general indications (symptomatic patients, recurrent hernias, bilateral hernias, etc.) and contraindications (no absolute but some relative contraindications) should be carefully assessed and mentioned. The procedure should be performed step-by-step. Entering the preperitoneal space, trocar placement, and insufflation are very important and should be specified. Elaborate knowledge of the inguinal region, anterior abdominal wall, and preperitoneal space is paramount. The preperitoneal space, which lies between the transversalis fascia and the parietal peritoneum, contains areolar and adipose tissue and the inferior epigastric artery and vein. Other important parts of the preperitoneal space are the Cooper ligament and the iliopubic tract. Access to and good dissection of the preperitoneal region (space of Retzius) should be explained. A space maker can be used to access the preperitoneal area. Great efforts should be made to avoid peritoneal perforation. Violation of the peritoneum may cause loss of insufflation from the preperitoneal space into the peritoneal cavity, thereby resulting in a collapse of the preperitoneal space, making the procedure more difficult and leading to intraabdominal organ injury. The surgeon must be aware of the triangle of pain containing important nerves, the triangle of doom containing the external iliac artery and vein, and other vascular structures. Mesh tacking should be avoided in these areas.

## Conclusions

In conclusion, simulation-based training systems and websites may help decrease both the time for the learning curve and the anxiety caused due to the risk of encountering a surgical complication during the training period. However, as can be understood from our study, academic platforms containing TEP videos need further improvement in terms of the educational quality for innovative surgical techniques. We believe TEP videos should be standardized according to TepSS points, that each step should be explained carefully, and that the videos should be uploaded after a careful peer-review process.
